# Integrative analysis of Multiple Sclerosis using a systems biology approach

**DOI:** 10.1038/s41598-018-24032-8

**Published:** 2018-04-04

**Authors:** Karla Cervantes-Gracia, Holger Husi

**Affiliations:** 1 University of Monterrey, Health Sciences Division, Monterrey, 66238 Mexico; 2 Institute of Cardiovascular and Medical Sciences, BHF Glasgow Cardiovascular Research Centre, University of Glasgow, Glasgow, G12 8TA UK; 3 Department of Diabetes and Cardiovascular Science, University of the Highlands and Islands, Centre for Health Science, Inverness, IV2 3JH UK

## Abstract

Multiple sclerosis (MS) is a chronic autoimmune disorder characterized by inflammatory-demyelinating events in the central nervous system. Despite more than 40 years of MS research its aetiology remains unknown. This study aims to identify the most frequently reported and consistently regulated molecules in MS in order to generate molecular interaction networks and thereby leading to the identification of deregulated processes and pathways which could give an insight of the underlying molecular mechanisms of MS. Driven by an integrative systems biology approach, gene-expression profiling datasets were combined and stratified into “Non-treated” and “Treated” groups and additionally compared to other disease patterns. Molecular identifiers from dataset comparisons were matched to our Multiple Sclerosis database (MuScle; www.padb.org/muscle). From 5079 statistically significant molecules, correlation analysis within groups identified a panel of 16 high-confidence genes unique to the naïve MS phenotype, whereas the “Treated” group reflected a common pattern associated with autoimmune disease. Pathway and gene-ontology clustering identified the Interferon gamma signalling pathway as the most relevant amongst all significant molecules, and viral infections as the most likely cause of all down-stream events observed. This hypothesis-free approach revealed the most significant molecular events amongst different MS phenotypes which can be used for further detailed studies.

## Introduction

Multiple sclerosis (MS) is considered the leading cause of non-traumatic disability worldwide in young adults. MS is a long-lasting inflammatory disorder characterized by lesions in the central nervous system that give rise to progressive neurodegeneration. Globally, 2.3 million people were reported to have MS in 2013 and the overall trend is for an increase of its incidence and prevalence amongst the general population^1^. MS prevalence varies among gender and ethnicity, the highest incidence is found in North America and Europe with a ratio of 2:1 women to men affected with the disease. Additionally, the economic burden of MS treatment and management is constantly rising, with a total yearly cost of approximately 40, 000 USD per patient estimated in 2007 as a global average^2^.

Clinically isolated syndrome (CIS) is recognized as an initial single episode of inflammatory demyelination, from which 30–70% progress to MS. CIS is the first clinical presentation of the disease in 85% of MS patients^3^. During the initiation of the disease, relapse events of focal inflammatory lesions of the white matter can be detected; relapse events are commonly followed by a complete/partial recovery of the lesion. As the disease progresses, an expansion of the pre-existing lesions occurs, leading to a high level of neurodegeneration^4^. The International Advisory Committee on clinical trials in MS has defined a phenotype classification system. The most common MS phenotype is called RRMS (relapsing-remitting MS) and accounts for 85% of MS cases. RRMS is characterized by the initiation of MS events and 75% of these cases evolve into a progressive level called SPMS (secondary-progressive MS), where no remission can be observed and continuous neurodegeneration happens. Primary-progressive MS (PPMS) accounts for approximately 15% of MS cases. PPMS is characterized by the progressive accumulation of disability from onset, usually without any remission events. PPMS cases that present recovery events are called primary-remitting MS (PRMS)^5^.

**Figure 1:**
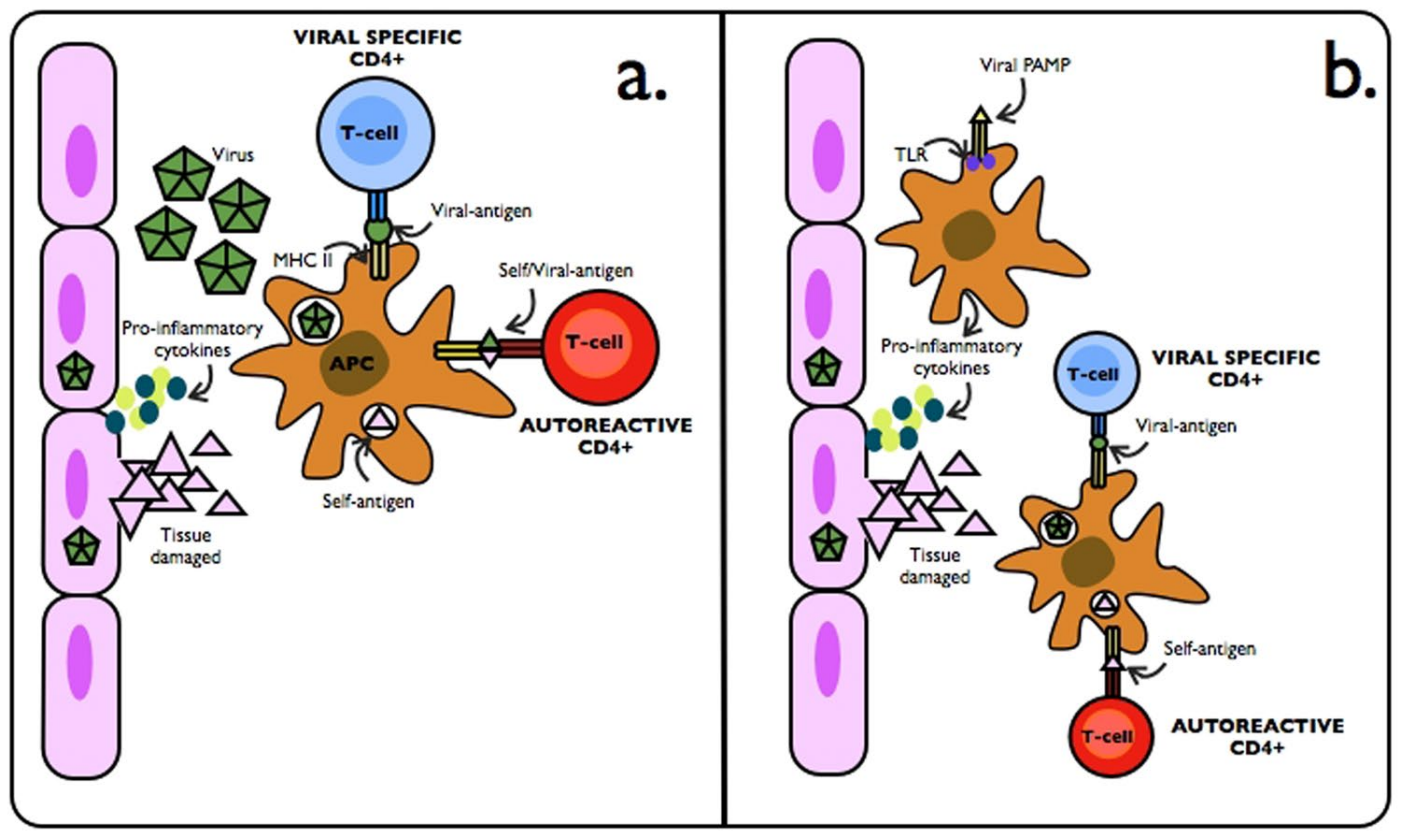
Representation of molecular mimicry and bystander activation mechanisms. Molecular mimicry (**a**) is defined where pathogen and self-antigens are believed to share epitopes, and antigen-cross reaction can occur through T cell receptors (TCR**)**. Bystander activation (**b**) can also reach CNS autoimmunity. In this process, pathogen or endogenous ligands can activate APCs through Toll-like receptors (TLRs), stimulating APCs production of pro-inflammatory cytokines (i.e. IL1, IL6, IL12), over- differentiation and activation of autoreactive T-cells.

Although the nature of MS remains elusive, it’s been reported that MS imply a complex interaction between environmental and genetic factors. Several environmental factors have been identified to play a role in MS, including lack of vitamin D, smoking and Epstein-Barr virus exposure. Concerning genetic factors, the HLA-DR*1501 variant is considered the major genetic susceptibility factor. However, most of the genetic factors associated with MS are believed to have low to moderate impact^6^. Aiming for a better understanding of MS genesis, mechanisms like mimicry and bystander activation have caught attention (Fig. 1)^7^, from which the activation of autoreactive T-cells and subsequently central nervous system (CNS) autoimmunity can be explained.

**Figure 2:**
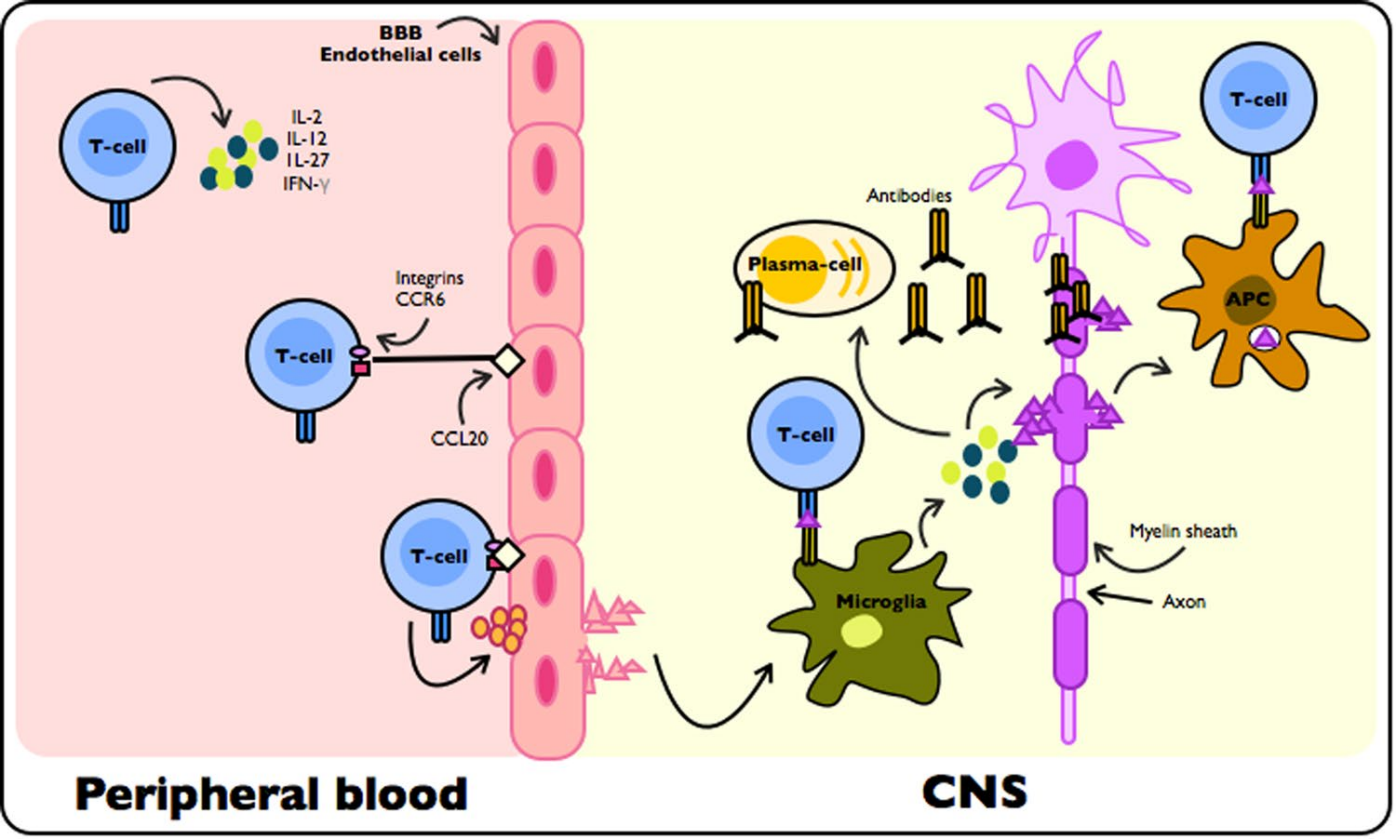
Suggested mechanisms for MS pathogenesis. Through peripheral activation of specific Th1 cells and subsequent release of pro-inflammatory cytokines (IL-1, IFN- γ, TNF, IL-12, IL-17), ligands (i.e. CCR6, integrins) on T-cell surface and adhesion molecules of BBB endothelial cells (i.e. CCL20) are activated. This event allows T-cell – BBB binding and BBB breakdown by matrix metalloproteases secreted by T-cells. This event triggers the recruitment of other autoimmune cells. Once in the CNS T-cells secrete pro-inflammatory cytokines, activate secretion of chemokines through microglia/macrophages and recruit more autoimmune cells. Myelin specific T-cells are thought to be activated through APCs by antigen- HLAII molecule recognition and co-stimulatory molecules, which are activated by soluble proteins of degraded myelin and spread its epitopes. These events lead to myelin destruction and neurodegeneration.

Specific myelin detection and its subsequent damage, is due to myelin specific T-cells that are primary effectors in MS pathogenesis^8^. Myelin specific T-cells are thought to identify self-antigens, containing the signature of the myelin sheets. MOG, PLP and MAG are some myelin molecules that have been reported to share peptides with pathogens, reason why these autoreactive T-cells are thought to be involved in MS development and progression^8^. These T-cells are believed to be activated in the periphery by environmental triggers processed by antigen presenting cells (APCs), such as microglia, macrophages, dendritic cells and B cells, and are detected by autoreactive T-cells in complex with genetic factors, following the infiltration of immune cells into the CNS through the blood-brain barrier (BBB) and initiation of MS immunopathology (Fig. 2).

Monozygotic twin studies have shown an MS susceptibility rate of around 30%^9^, giving an insight of the increment of MS severity through a direct environmental impact on the genetic background. The most significant genes highlighted through genome-wide association studies (GWAS) are related to the HLA class II gene cluster displaying clear linkage disequilibrium. Moreover, meta-analysis and follow-up of several MS GWAS studies have led to a convincing association of these variants detected with MS. As for class I allele variations, HLA-A*0201 shows a protective effect, whereas several other class II alleles confer MS susceptibility^10^. Among non-HLA significant gene variations associated with MS susceptibility are IL2RA, ILRA7, CYP27B1, MGAT5, MGAT1, STAT3, CD40, IRF8, and others. Despite the information that GWAS have provided, it is not completely clear how these variations give MS susceptibility^11^.

Hypothesis-driven approaches have predominated in the search of MS aetiology, albeit with limited success. Although GWAS analyses are non-biased approaches, an in-depth data analysis is still lacking, and challenges such as replication, sample size and source lead to a poor understanding of the key molecules and processes involved in MS progression. The “recent” implementation of integrative approaches for the understanding of complex disorders has led to the rise of un-biased new hypotheses to explain diseases. The analysis and integration of a huge amount of data result in the identification of conserved networks significantly deregulated in a determined disorder (hypothesis) across studies, and can be further validated through traditional procedures (Fig. 1). This approach is called systems biology and in contrast to traditional methodologies, multiple significant molecules from numerous studies (i.e. GWAS, gene expression profiling, proteomics) are integrated and simultaneously analysed. By combination of different data it is possible to uncover hidden patterns in datasets giving rise to network interaction models, and clearer insights of the mechanisms involved in complex diseases^4,5^.

This work implements a systems biology approach to produce a clearer view of the deregulated molecular mechanisms involved in MS. As an initial framework a Multiple Sclerosis (MuScle) database was constructed to help in data management and retrieval. This also gives the possibility to exchange and reuse this data in further analyses. An integrative analysis of multiple gene expression profile studies from PBMC of healthy donors, MS patients and MS treated patients was performed with publicly available data, by applying statistical filters, specific systems biological pipelines and visualisation tools.

## Results

GEO searches resulted in 18 datasets (Supplemental Table S1) that were retrieved, including mRNA expression and miRNA expression profiles. Several comparisons within each dataset were performed due to their high heterogeneity (i.e. healthy donors, RRMS, SPMS, PPMS, different treatments regimens) resulting in a total of 44 dataset comparisons (Supplemental Table S2). Additionally, we compared our results with differentially expressed genes resulting from Epstein-Barr virus (EBV) infected peripheral blood, inflammatory conditions due to Mycobacterium tuberculosis (TB) infection, induced allergic reactions and sarcoidosis, and autoimmune responses observed in peripheral blood of patients suffering from systemic lupus erythematosus (SLE), rheumatoid arthritis (RA), juvenile idiopathic arthritis (JIA) and enthesitis-related arthritis (ERA).

**Figure 3:**
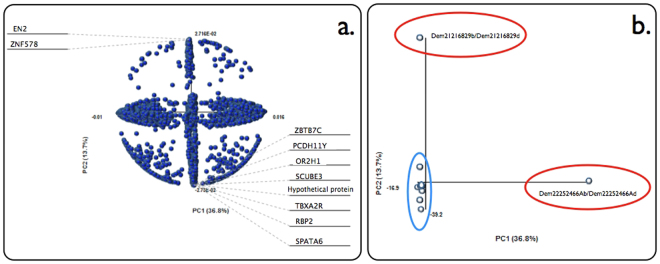
Principal component analysis of all genes and datasets used in this study. The highlighted molecules in the variable plot (**a**) are the most significant ones that influence the model and how they are correlated to each other. The samples plot (**b**) is a graphical representation of the datasets, showing Dem21216829b/Dem21216829d and Dem22252466Ab/Dem22252466Ad datasets were the most dissimilar ones and were removed.

Significance p-value cut-offs (<0.05) were applied to the results of the statistical analyses obtained through GEO2R, and log 2 fold-change (FC) cut-offs (log2FC >1 and <−1) to the interrelated dataset groups stratified by PCA (Fig. 3), resulting in a total of 35 dataset comparisons. From the “General” group of datasets, 5079 significant up and down-regulated molecules were identified. The “Non-treated” group consisted of 13 dataset comparisons, from which 16 up and down-regulated molecules were shared in more than 20% of all comparisons in this group (Table 1). The most significant gene is the ankyrin repeat domain-containing protein 10 (ANKRD10) with a 31% of up-regulated frequency among the dataset comparisons. Of these 16 molecules, three were also found to be statistically significant in naïve EBV infected peripheral blood after applying thresholding parameters, namely C1ORF228, CNTNAP2 and LPP. However two of the three entries showed an opposite trend in differential expression between case and control. Further comparison with differentially expressed genes relating to inflammatory responses showed two molecules of these 16 with similar fold-change trends (CNTNAP2 and PDE4DIP), and a comparison with autoimmune disease states resulted in an overlap of 6 genes (SCL8A1, MALAT1, C1ORF228, CNTAP2, RIF1 and SMAD4), though 3 of these (SCL8A1, CNTAP2 and RIF1) were modulated in the opposite direction compared to MS.

**Table 1:** Most significant, correlated and deregulated molecules without treatment.

CluSO	Gene	Name	“Non-treated” group (n = 13)		Virus infected group (n = 2)		Inflammation group (n = 3)		Autoimmune group (n = 14)	
			Regulation	logFC^a^	Regulation	logFC^a^	Regulation	logFC^a^	Regulation	logFC^a^
B0717	ANKRD10	Ankyrin repeat domain protein 10	Up	2.21 (4)		ns^b^		ns^b^		ns^b^
B0384	ADD2	Beta- adducin	Up	2.12 (3)		ns^b^		ns^b^		ns^b^
BA289	SLC8A1	Sodium/calcium exchanger 1 precursor	Up	3.11 (3)*		ns^b^		ns^b^	Down	−1.24 (1)
B9374	MALAT1	Metastasis associated lung adenocarcinoma transcript 1 protein	Down	−1.92 (3)*		ns^b^		ns^b^	Down	−1.66 (2)
B1814	C1ORF228	Uncharacterized protein C1ORF228	Up	1.28 (3)	Down	−1.27 (1)		ns^b^	Up	1.66 (1)
BF644	SSTR2	Somatostatin receptor type 2	Down	−2.32 (3)		ns^b^		ns^b^		ns^b^
B5523	F5	Coagulation factor V precursor	Down	−1.4 (3)*		ns^b^		ns^b^		ns^b^
B3266	CNTNAP2	Contactin- associated protein-like 2 precursor	Down	−3.21 (3)*	Down	−1.25 (1)	Down	−1.08 (1)	Up	2.03 (1)
BG121	TAF9B	Transcription initiation factor TFIID subunit 9B	Up	1.91 (3)		ns^b^		ns^b^		ns^b^
B9009	LPP	Lipoma preferred partner	Up	1.48 (3)*	Down	−1.64 (1)		ns^b^		ns^b^
BB904	PAWR	Prostate apoptosis response protein PAR-4	Down	−2.09 (3)		ns^b^		ns^b^		ns^b^
BD727	RIF1	Telomere-associated protein RIF1	Down	−1.64 (3)		ns^b^		ns^b^	Up	1.38 (1)
BA240	PDE4DIP	Myomegalin	Up	1.27 (3)		ns^b^	Up	1.11 (1)		ns^b^
BF121	SMAD4	Mothers against decapentaplegic homolog 4	Up	1.56 (3)		ns^b^		ns^b^	Up	1.2 (1)
BJ537	DKK3	Dickkopf-related protein 3	Up	1.86 (3)		ns^b^		ns^b^		ns^b^
B9548	MDM4	MDM4 P53 binding protein homolog	Up	1.58 (3)*		ns^b^		ns^b^		ns^b^

*Molecules found to also be regulated in the opposite direction, shown in a single dataset; ^a^Number in brackets denote the number of significant occurrences in all samples; ^b^Non-significant.

The remaining 22 datasets, the “Treated” group, contains a cluster of 16 of the most significant molecules that are shown in Table 2. Within the 22 dataset comparisons radical S-adenosyl methionine domain-containing protein 2 (RSAD2) and interferon-induced GTP-binding protein Mx1 (MX1) were the most significant molecules with a frequency of at least 50% to be up-regulated across this dataset group. The results in Table 2 were also contextualised by comparison to data derived from EBV infected peripheral blood samples. Four (IFI27, ISG15, SIGLEC1 and EPSTI1) out of the 16 molecules were also found to be indicative of EBV infection, also showing the same trend in differential expression between case and control. The comparative analysis with inflammation showed an overlap of three molecules (RSAD2, PLSCR1 and EPSTI1), whereas the autoimmune disease group displayed a complete overlap with the significant molecules associated with treated MS patient blood samples, both in terms of fold-change directionality and similar fold changes apart from EIF2AK2, which displayed either an up- or down-regulation in 50% of all significant data points.

**Table 2:** Most significant, correlated and deregulated molecules after treatment.

CluSO	Gene	Name	“Treated” group (n = 22)		Virus infected group (n = 2)		Inflammation group (n = 3)		Autoimmune group (n = 14)	
			Regulation	logFC^a^	Regulation	logFC^a^	Regulation	logFC^a^	Regulation	logFC^a^
BE141	RSAD2	Radical S-Adenosyl methionine domain-containing protein 2	Up	2.52 (14)*		ns^b^	Up	1.35 (1)	Up	2.36 (4)
BA153	MX1	Interferon-induced GTP-binding protein MX1	Up	1.92 (11)*		ns^b^		ns^b^	Up	1.66 (4)
B7528	IFI44L	Interferon-induced protein 44-like	Up	2.99 (10)*		ns^b^		ns^b^	Up	2.25 (7)
B7546	IFI27	Interferon-alpha inducible protein 27	Up	4.05 (10)*	Up	2.25 (2)		ns^b^	Up	4.04 (5)
BI124	BIRC4BP	XIAP associated factor-1	Up	1.54 (10)*		ns^b^		ns^b^	Up	1.54 (3)
B7105	HERC5	Probable E3 ubiquitin-protein ligase HERC5	Up	1.96 (10)*		ns^b^		ns^b^	Up	1.73 (6)
B7896	ISG15	Ubiquitin-like protein ISG15	Up	2.15 (10)*	Up	1.33 (1)		ns^b^	Up	1.98 (6)
BF209	SIGLEC1	Sialoadhesin precursor	Up	2.83 (10)*	Up	1.95 (1)		ns^b^	Up	2.88 (2)
BF513	SPATS2L	SPATS2-like protein	Up	1.8 (9)*		ns^b^		ns^b^	Up	1.76 (4)
BC522	PLSCR1	Phospholipid scramblase 1	Up	1.32 (9)*		ns^b^	Up	1.10 (2)	Up	1.58 (1)
BH558	USP18	Ubl carboxyl-terminal hydrolase 18	Up	2.58 (9)*		ns^b^		ns^b^	Up	2.41 (4)
B4753	EIF2AK2	Eukaryotic translation initiation factor 2-alpha kinase 2	Up	1.35 (9)*		ns^b^		ns^b^	Up	0.15 (4)**
B7547	IFI44	Interferon-induced protein 44	Up	1.69 (9)*		ns^b^		ns^b^	Up	1.88 (5)
B7549	IFIH1	Interferon-induced helicase C domain-containing protein 1	Up	1.44 (9)*		ns^b^		ns^b^	Up	1.1 (4)
BB257	OAS2	2′-5′-oligoadenylate synthase 2	Up	1.56 (9)*		ns^b^		ns^b^	Up	1.63 (5)
B5174	EPSTI1	Epithelial stromal interaction protein 1	Up	1.69 (9)*	Up	1.26 (1)	Up	1.12 (2)	Up	2 (2)

*Molecules found to also be regulated in the opposite direction, shown in a single dataset; **Molecules found to also be regulated in the opposite direction, shown in 2 datasets; ^a^Number in brackets denote the number of significant occurrences in all samples; ^b^Non-significant.

**Figure 4:**
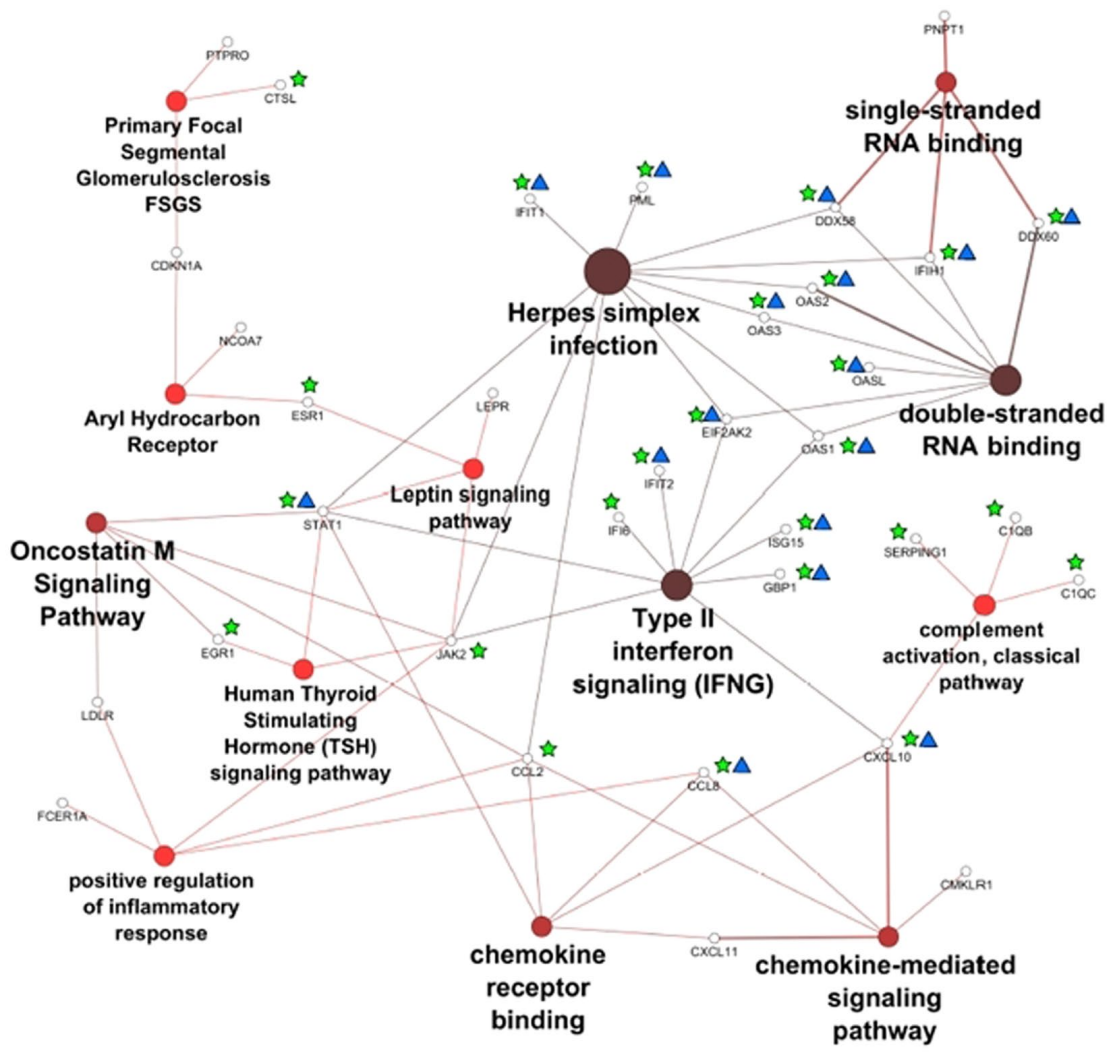
Processes and pathways identified within the most significant molecules of the datasets. Gene ontology and pathway analysis was performed using significant molecules across all datasets. Node size reflects the number of associated molecules, and node colour corresponds to statistical significance. The darker the pathway node, the more statistically significant it is, with a gradient from red (p-value 0.05–0.005) to black (p-value < 0.0005). Green stars mark the molecules that were also statistically significant associated with innate immune response, and blue triangles denote statistically significant virus response molecules. Both latter biological function groups were removed from the figure to improve visualisation.

ClueGO analyses were performed in order to identify the relation within the statistically significant molecules in terms of overlapping or shared pathways and gene ontologies (Fig. 4). The most significant processes that showed an inter-connectivity between the most significant molecules with a frequency of at least 20% within all the dataset comparisons were chemokine activity, double-stranded RNA binding and single-stranded RNA binding gene ontologies, and most prominently the Interferon gamma (IFN- γ) signalling pathway, encompassing C-X-C motif chemokine 10 (CXCL10), 2′-5′-oligoadenylate synthase 1 (OAS1), ubiquitin-like protein ISG15 (ISG15), guanylate-binding protein (GBP1), interferon-induced protein with tetratricopeptide repeats 2 (IFIT2), interferon alpha-inducible protein 6 (IFI6), and interferon-induced, double-stranded RNA-activated protein kinase (EIF2AK2). All the molecules included in this analysis were up-regulated.

**Figure 5:**
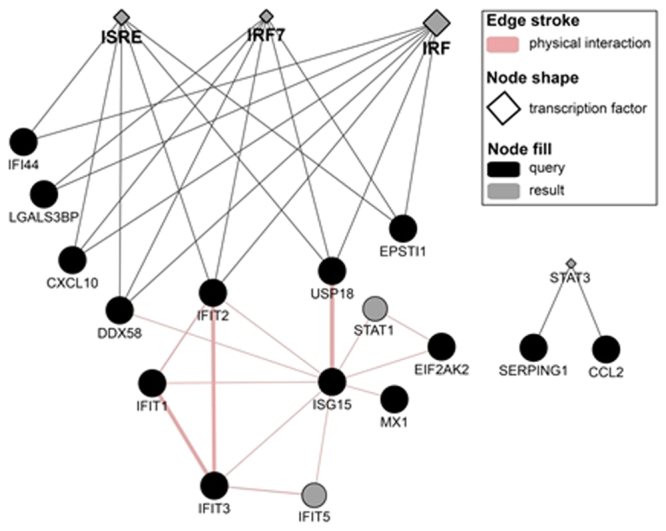
Protein-protein interactions and transcription factors associated with main molecules of the study. Three transcription factor sites were identified in IFI44, LGALS3BP, CXCL10, DDX58, IFIT2, USP18, EPST11; all three are transcription factors are related with IFN signalling. An additional stat3 transcription factor site was found in SERPING1 and CCL2. Pink connections represent the physical interaction of molecules.

Lowering of threshold parameters for further exploratory analysis resulted in a total of 97 molecules that were included in the subsequent steps with the lowest frequency of 11% within all the dataset comparisons. GO and pathway clustering (Fig. 4) showed that the tyrosine-protein kinase JAK2 (JAK2) and the signal transducer and activator of transcription 1-alpha/beta (STAT1) were the genes more involved and shared among the pathways found. Separately analyses of the groups were performed. The “treated” group had similar results as the general analysis. As for the “non-treated” group, no significant gene ontologies associations neither pathways could be determined. The following pathway analyses were based on the general molecules and the “treated” ones. Further analysis using GeneMania showed three transcription factors (ISRE, IRF7 and IRF) associated with IFN signalling, and STAT3 with Toll-like receptor (TLR) signalling. Physical interactions (protein-protein interactions) were found between IFIT1, IFIT2, IFIT3, IFIT5, ISG15, USP18, EIF2AK2, MX1 and STAT1 (Fig. 5). After the association of the molecules from the dataset comparisons to a main pathway through term clustering in ClueGO, the next iteration step involved analysis of pathway statistical inference and visualisation in PathVisio. The signalling pathway of IFN- γ was shown to be the most significant one either in ClueGO and PathVisio analyses. Once the pathway was selected, it was then fully filled by the total significant molecules (5079) of the study into the software in order to visualise consistencies and inconsistencies in signal cascades and other chained events. Inconsistent pathways and sub-cascades were removed from pathway maps and excluded in the following de-novo pathway mapping.

**Figure 6:**
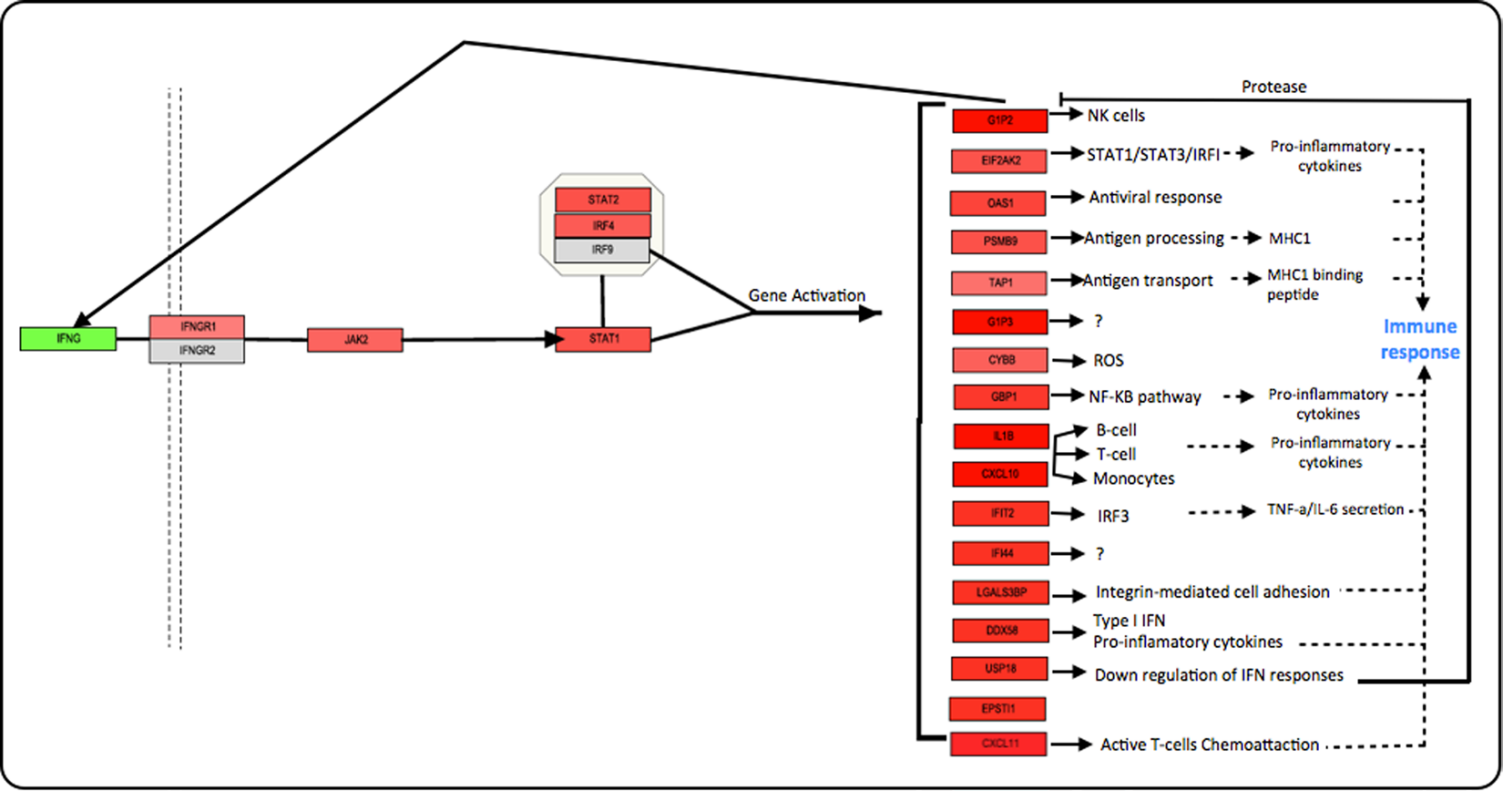
MS IFN- γ signalling pathway. IFN-γ pathway has been identified as the main pathway involved generally in this study through PathVisio analyses. Lines without an arrow mean binding; complete arrows mean activation/induction and dashed arrows mean activation through a unknown/more complex process. For the genes with question marks “?”, their function remains unclear. The up and down-regulated genes are represented in red and Green boxes respectively. The more strong the colour, the more up/down-regulated the gene. IFN-γ is down-regulated while the rest of the molecules in the pathway up-regulated possibly due to a feedback process with the molecules it induces.

The original pathway map of the IFN-γ signalling sourced from WikiPathways and displayed in PathVisio was redesigned, in order to fit the molecular relationships of the data and to coherently contextualise the clinical presentations of MS (Fig. 6). The IFN signalling cascade in MS is represented by up/down-regulated molecules of the study and lead to activation of genes associated with immune response induction. The pathway was enriched with the molecules that shared IFN TFs that were found through GeneMania. Here, a clear negative feedback loop can be observed, in which Ubiquitin-like protein ISG15 (G1P2) induces IFN-γ expression while Ubl carboxyl-terminal hydrolase 18 (USP18) inhibits G1P2 activity, explaining the observed down-regulation state of IFN-γ.

**Figure 7:**
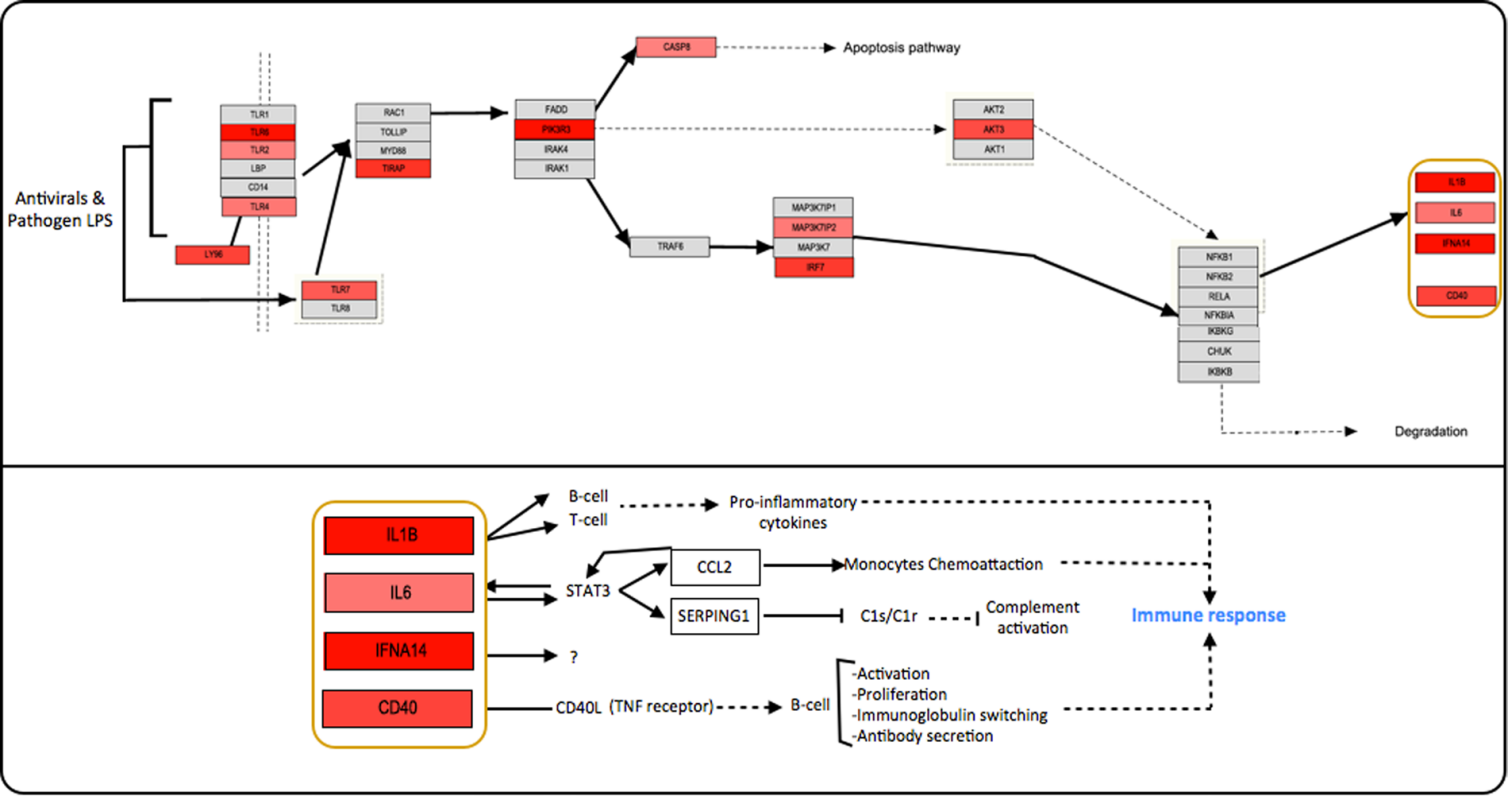
MS TLR signalling pathway. Toll-like receptor signalling pathway was identified through the treatment group molecules and filled with the entire set of significant molecules found in the study; it was personalized in PathVisio WikiPathways and complemented with GeneMania results. A brief explanation of the final products function is described. Arrows mean activation/induction; dashed arrows mean activation through a unknown/more complex process. The genes up-regulated in this study are represented in red boxes, the more red, the more up-regulation they present.

After the analysis in PathVisio of the “treated” group, the TLR signalling pathway (Fig. 7) was considered the most relevant one due to molecular consistency criteria and statistical significance with a high Z-score and permuted p-value of less than 0.05. Here, overexpression of proinflammatory molecules, Interleukin-1 beta (IL1B), Interleukin-6 (IL6) and Tumor necrosis factor receptor superfamily member 5 (CD40) can lead to T-cell, B-cell and monocyte activation and eventually production of immune response, possibly leading to pro-inflammation.

## Discussion

Based on prior knowledge, the objective of this study was to generate a better understanding regarding the molecular mechanisms underlying MS pathophysiology by analysing large-scale transcriptomics data and deciphering how the most significant molecules involved in the disease are affecting each other. Although all the individual gene expression profiles used in this study have been previously reported and analysed, a combinatorial integration of all the data at the systems level can ultimately yield to new biological insights over MS. Due to the high variability and uncertainty of molecular identifiers found within databases such as EnsEMBL and UniProt, in this project the PADB database was used (www.PADB.org) to harmonise and integrate molecular data. The PADB database was chosen due to its accuracy, great coverage and record of different identifiers for each molecule from different databases such as UniProt and EnsEMBL, and transcriptomics data platforms (i.e. Affymetrix, Illumina, Agilent).

By analysing the results obtained in the group of “Non-treated” molecules and as a control of our findings, it was identified that some of the significant genes up/down-regulated shown in Table 1 have been previously associated with MS. C1ORF228, LPP, SSTR2, SLC8A1, DKK3 and SMAD4 have been associated with MS autoimmune response events, i.e. SSTR2 is a receptor for the neuro-protective molecule ‘somatostatin’ which presents a gradual reduction in response to inflammation. The down-regulation of SSTR2 has been associated with high cytokine levels/inflammation and progression of the disease^12^. SMAD4 has also been associated with MS pathology, and it was reported that IL10 production from Th1 cells under induction of TGF-β via SMAD4 does occur^13^. IL10 is an anti-inflammatory cytokine that inhibits Th1-effector functions and it is usually increased during remission. Its expression can also be induced by MS treatments (i.e. IFNB, glatiramer acetate)^14^. This event might be a possible explanation of the remission stages, where Th1 effector cells produce their own inhibitors. Thus, the up-regulation of SMAD4 found in this analysis match and relate to IL10 overexpression. Another gene of interest identified in this study is DKK3. Its expression is essential to maintain T-cell controlled activation and to protect tissues from self-destructiveness. DKK3 down-regulation has been reported in the experimental autoimmune encephalomyelitis (EAE) animal model, where it leads to a local increment of CD8 + T-cell reactivity, which worsens the symptoms^15^.

The remaining molecules (Table 1) have not been associated with MS yet, however most of them have been related with synapsis and neuronal processes in the CNS and PBMCs. The most significant one, ANKRD10, is involved in the regulation of the canonical-Wnt signalling pathway. Among other roles, its pathway inhibition promotes myelination, and, when activated, delays the differentiation of precursors into myelin oligodendrocytes^16^. It is notable that generally all ankyrin repeat domain containing molecules have been shown to be involved in the formation of transcription complexes, the initiation of immune responses, biogenesis and the assembly of protein complexes also linked to cell adhesion, and it is conceivable that this particular molecule is an as yet unattributed key player in adhesion. In this analysis, ANKRD10 was observed to be up-regulated in MS samples, which can be related with a myelination arrest of MS. MALAT1 has also been related with regulation of neurodegeneration. The up-regulation of this molecule protects from retinal neurodegeneration, but in this study MALAT1 is down-regulated, therefore potentially associated with other MS symptoms^17^. Of particular interest is also the observed down-regulation of Contactin- associated protein-like 2 precursor (CNTNAP2), which was observed both in MS and EBV-infected samples. This molecule is a well-known adhesion molecule that belongs to the neurexin family and functions in the vertebrate nervous system as a cell adhesion molecule and receptor. It is localized at the juxtaparanodes of myelinated axons, where it mediates interactions between neurons and glia and has been implicated in many neurodegenerative processes, and is quite likely a key player in other neurodegenerative disorders such as MS.

As for the “Treated” group, most of the significant molecules identified through the statistical filters (Table 2) are consistent with previously reported investigations. A study describing biomarkers specific to IFN-β treatment activity identified RSAD2, MX1, IFI27, IFI44L, HERC5, SIGLEC1, USP18, EPSTI1 through gene expression profiles^18,19^. These molecules were confirmed to be activated by IFN-β when compared to IFN-γ activity. It was also determined that these molecule expressions were decreased in the presence of neutralizing antibodies (NAB). A different study reported similar findings by using an integrative approach, but following a different criteria and data sources (gene expression profiles, GWAS)^16,18^. Through these findings it can be established that by using a completely unbiased approach it was possible to reach the same outcome as previous studies.

Common gene expression analyses, GWAS, and so on, have problems supporting their results due to small number of samples and lack of replication. Here better results were obtained from the “Treated” group than from the “Non-treated”, this could be explained due to the sample size, source (PBMC) and the great disease heterogeneity. However, through the combination of large data sets from previous studies, these limitations were somehow diminished. Moreover, these results should be interpreted under the assumption that the sources of the data are PBMC samples, not CNS tissue. Further confirmation that the PBMC molecules found represent CNS events is needed.

Concerning the pathway analysis, it is pertinent to keep in mind that these were generated by the inclusion of all the dataset comparisons, where the most significant molecules belong to the treated group, simply due to the bigger size of experimental studies. Therefore, this is a potential weakness of the study. Since ClueGO could significantly assign neither gene ontologies nor pathways to the “Non-treated” molecules, and their Wikipathways in Pathvisio were inconsistent, these molecules were combined with the treated ones, leading to Figs 4–7. Instead of having a clean and consistent comparison without any external noise, here the final hypothesis could be a reflection of the treatments or the pathogenesis of MS. Further analysis should be performed, including higher quantities of gene expression profiles, the inclusion of omic’s studies and detailed analyses.

For more than 30 years IFN-γ has been reported as one of the major proinflammatory cytokines found in MS, however its role remains uncertain. IFN-γ secretion is thought to be Th1 cells hallmark. NK-cells and APCs also produce IFN-γ, which binds to almost all cell types due to its ubiquitously expressed receptor. This double role needs to be studied in more depth^20,21^. Here IFN-γ signalling pathway was the most significant and consistent among all the significant molecules of the study. It was considered as the focus-signalling pathway. GeneMania results of TFs shared within the molecules showed that most of the molecules involved in this pathway lead to immune response. Some gene products can also be described in the IFN-β-signalling pathway. It was shown that the signalling and induction of differently expressed genes depends on the cell types, finding a correlation within each other to activate the main factors (i.e. cytokines, chemokines) mainly found in MS patients^19,22^. It has also being reported that besides viral induction, IFN-γ T-cells can be activated through IFN-α or β, leading to IFN-γ signalling^19^. Further studies need to be done to identify which agents can activate IFN-γ expression. The observed down-regulation can also be studied by, for example, investigating USP18 activity and whether it can be associated further to a specific cell type and stage of the disease.

Although the main cell types that take place in CNS neurodegeneration have been previously described, their precise mechanism is unknown. Moreover, it has been previously reported that IFN-γ influence the expression of different molecules in each cell type involve in MS, which can be associated with its dual role in the disease^20^. Since in some of the studies included in this analysis several cell types were isolated (CD4+, CD8+, CD34+), Fig. 6 represents a global but specific outcome of IFN-γ induction within different PBMCs and further analysis of the significant molecules found in this study can be performed.

Several hypotheses can rise from this pathway, where every up-regulated molecule can be individually validated. As an example, the overexpression of specific chemokines and cytokines IL1B, CXCL10, CXCL11 that activate microglia/macrophages, T-cells, B-cells, and others can be compared within MS phenotypes, and ascertain if PBMCs can mirror CNS activity. Validation of the significant molecules can include the identification of the cell type that produces them, as well as abundance in the CNS of MS patients. These molecules can be compared within MS phenotypes to see if there is an expression difference, and a correlation with remission or progression of the disease can be reached.

Additionally, the over-representation of the TLR signalling pathway can be associated to MS treatment. The significance of this pathway within the “Treated” group of molecules matches the definition of the pathway, where its activation is induced through antivirals^21^. This result shows a probable mis-target of pro-inflammatory molecules induced by treatment (i.e. IFN-β). The validation of these molecules as biomarkers of treatment activity and productivity can also be proposed.

**Figure 8:**
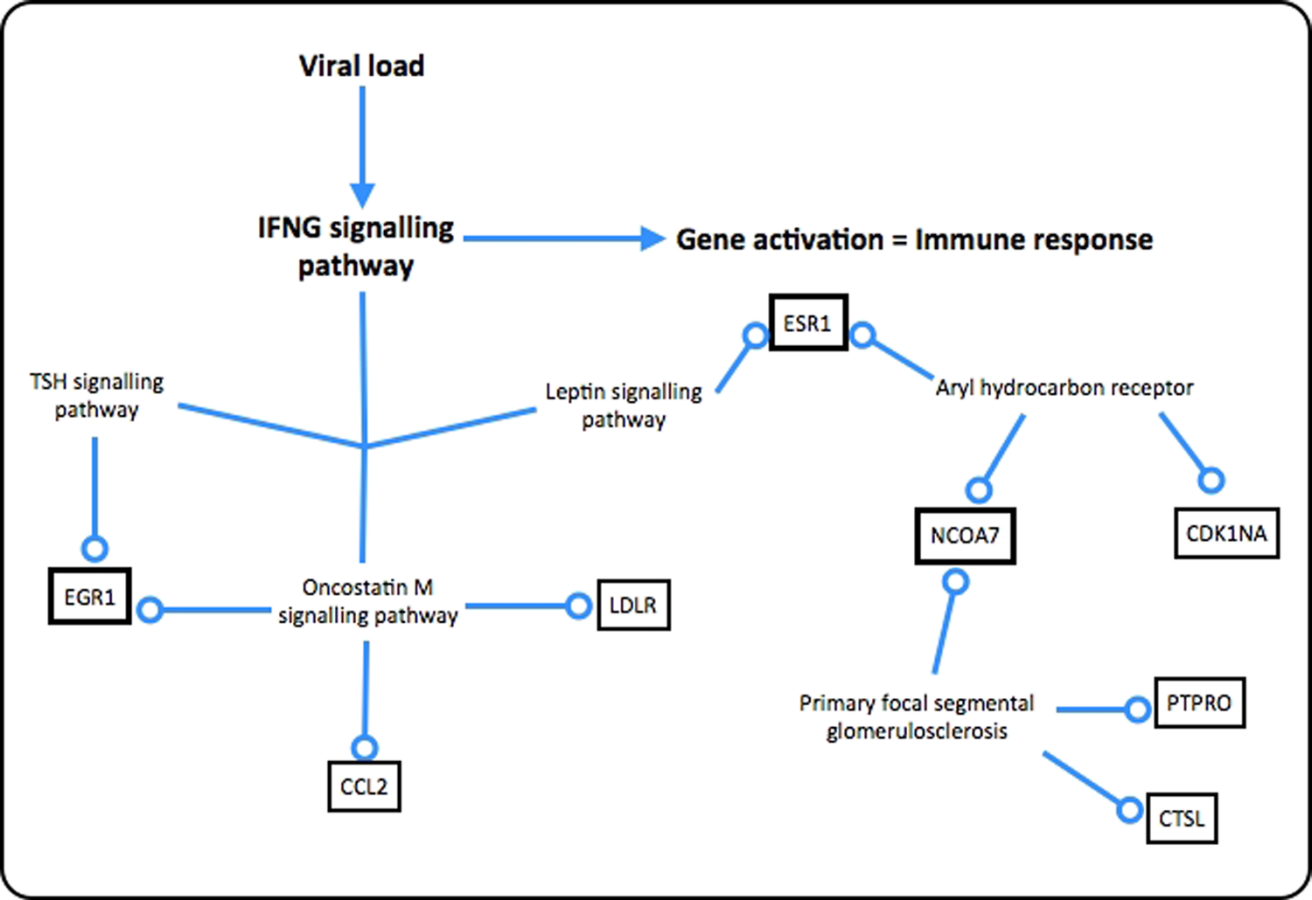
Multiple sclerosis pathway. IFN-γ signalling is activated by viral load and produced immune response; other pathways are thought to be involved in the significant gene response, however pathway names can be unrelated with MS at all. Gene ontologies can be highlighted just for those pathways were those genes have been identified.

Taking into account the different analyses carried out, a final hypothesis was generated. Through ClueGO pathway analyses and PathVisio validation, IFN-γ is considered the main pathway involved in MS. An activation of this pathway through viral load was properly identified by the significant involvement of antiviral pathways (Fig. 4). Although a common immune response outcome is expected, the correlation of other pathways was also detected. Every pathway associated with the most significant genes (Fig. 8) was previously related with MS pathophysiology. Oncostatin M has a known role in MS due to the up-regulation of Intercellular adhesion molecule-1 (ICAM1) in CNS epithelial cells^22^. It also induces pro-inflammatory IL-6 expression^23^. Primary focal segmental glomerulosclerosis (FSGS) has been reported to be induced by IFN-β and γ treatment^24^. Aryl hydrocarbon receptor has also been associated with MS and is gaining focus due to the association of the gut microbiota composition and MS^25^. AHR has been reported as linked with Th17 and T-regulatory (T-reg) cells and thus, involved in immune response regulation, and as for autoimmune diseases, AHR can supress it or make it worse, depending on its ligand^26^. In order to validate and in this way corroborate the results of this study a follow-up experimental setup would be necessary.

In order to further contextualise and corroborate the findings in light of the findings regarding the potential upstream effector of EBV infection, we compared the observed expression profiles of statistically significant and across-dataset correlated molecules to naïve datasets relating only to EBV-infections and peripheral blood. A comparison of the most significant molecules involved in MS pathophysiology (Table 1) shows little overlap of only three molecules concerning significance, however the dysregulation between case and control of two of these three molecules demonstrate a completely opposite trend between MS and EBV infection. This profile between MS and EBV is slightly more correlated in the treatment group (4 out of 16 molecules with same dysregulation directionality) as shown in Table 2, which is somewhat expected given the target of the pharmacological treatment. Therefore, the observed common molecular pattern in MS, as a result of an upstream event of EBV infection, is indeed specific to MS pathophysiology and does not reflect the primary infection per se. This is likely due to a response further downstream to the infection, and an apparent viral infection by EBV is not the direct causative agent of the changes we observed. Yet there is mounting evidence that MS is tightly linked to EBV infection, whereby viral expression was observed in the CNS of MS cases^27,28^. It is also of importance to note that EBV is capable to express latent endogenous retroviral genes in the human genome, some of which are pro-inflammatory and have super-antigenic properties and have been shown to cause neurotoxic effects^29^. It is not possible to state whether EBV infection is the sole contributor or initiator of observed MS-based molecular changes in blood based on our analysis, but the literature suggests that multiple health-affecting causes are contributing factors to MS pathophysiology and/or initiation events, of which EBV is one of them.

As mentioned above, inflammation and inflammatory responses are intricately linked to the MS pathophysiology^30,31^. Therefore the MS-derived data was compared with a sarcoidosis dataset, primarily since it was reported that prolonged interferon beta treatment of MS patients can induce sarcoidosis^32,33^. Additionally, a dataset derived from allergic reaction was also used as another comparator due to its well characterized panel of modulated molecules in blood in inflammatory response^34^, as well as a comparison to infection by TB, which is well known to induce inflammatory effects observable in peripheral blood^35^. The cross-comparison between the significant MS data and a general inflammation status showed no considerable or consistent correlation with our observations. Only 5 molecules (CNTNAP2, PDE4DIP, RSAD2, PLSCR1 and EPSTI1) out of 32 showed a significance and similar fold-change trend compared to the MS data. PLSCR1 and members of this gene family were shown to be activated during acute phase responses^36^, and EPSTI1 has been reported to be involved in inflammatory gene expression regulation in macrophages^37^, but no substantial overlap between inflammatory conditions and MS could be demonstrated within the MS-specific molecules. Therefore, it is reasonable to assume that the correlated and statistically significant molecules presented in this study do not represent an inflammatory status observable in other investigations.

Another, and very important, facet regarding the aetiology of MS is the well described autoimmune disease aspect. Therefore the data derived from the MS analysis was compared to specific other autoimmune diseases, namely SLE, RA, JIA and ERA. SLE is a multisystemic autoimmune disease that can manifest itself in numerous pathological outcomes affecting skin, joints, lung, kidney and the CNS^38^. Indeed, it was reported in the literature that demyelinating disease in SLE in particular has the propensity to be accidentally misdiagnosed as other CNS affecting diseases such as MS^39^, and a case report showed an induction of SLE by interferon beta-1 treatment of a MS patient^40^, thereby demonstrating the potential autoimmune-link between these two diseases. RA, JIA and ERA are joint-affecting autoimmune disorders and are, like SLE, underpinned by chronic inflammation^41^. Large-scale GEO data retrieval and statistical analyses, followed by correlation to the MS results showed an overlap of 6 out of the 16 MS-specific molecules in the untreated MS-group, though 50% of these molecules (SCL8A1, CNTAP2 and RIF1) displayed an opposite fold-change in the case-control setting, and the other half (MALAT1, C1ORF228 and SMAD4) the same modulated directionality in the disease condition. This marginal overlap, confounded by dissimilar dysregulation, demonstrates to a considerable extent the specific nature of the MS-associated significant molecules which are distinct from modulated events observable in an autoimmune condition. However, a comparison and correlation of the autoimmune disease datasets with the treated MS-group shows a 100% correlation and association between both. This result shows that the molecular profile obtained from the treated MS-group is indistinguishable from the autoimmune condition group, and thereby constitutes the generic molecular response of MS disease pathophysiology observable in blood samples. It is quite feasible that this generic response has been “hidden” or overshadowed by the specific response observed in the untreated samples, especially since it is extremely unlikely that pharmacological treatment of MS patients would result in a molecular phenotype resembling or mimicking an autoimmune disease.

Additionally, to link autoimmune disease to viral attack, it was reported that EBV infection correlates epidemiologically with MS, SLE and RA, though the mechanism is as yet unknown, and whether EBV is a causative agent or to what level it contributes to an autoimmune response is still under debate^42^.

In summary, based on the large-scale data and molecular correlation analysis shown here, viral infection as well as inflammation are not the primary cause of molecular changes observed in MS blood samples. It can also be reported that key molecules, consistent and statistically significant, associated with untreated MS blood samples do not reflect a molecular composition in terms of gene modulation of autoimmune disease (mainly SLE). Yet there is clear evidence that pharmacological treatment of MS with appropriate compounds results in a molecular modulated pattern of specific, statistically significant and correlated molecules overlapping with the pattern observed in autoimmune disease. This might be due to “unmasking” by removal of MS-associated specific molecular changes observed in the untreated group after disease treatment. This would also explain why an overlap between the most consistent and statistically significant molecules in both MS untreated and treated analyses was not observed, whereby MS-specific responses can be attributed to the modulated changes of molecules listed in Table 1, and generic responses, in particular auto-immune disease, is reflected by the panel of molecular changes shown in Table 2 of the manuscript. One possible mechanistic interpretation of these findings is that pharmacological treatment of MS patients with interferon beta silences specifically the effects caused by EBV infection, yet does not modulate the autoimmune response effect. This is substantiated by the findings that interferon beta induces anti-inflammatory cytokines and represses HERV^43^, whose expression is directly linked to the presence and activity of EBV.On matters of validation targets selection, both the most significant molecules of the “Non-treated”/“Treated” groups as well as the processes and molecules involved in the IFN-γ signalling pathway and in the final hypothesis can be considered as potential novel targets. These molecules can be evaluated within MS patients with different phenotypes (CIS, RRMS, SPMS, PPMS), and a specific interval of time, in order to establish a possible link between molecules, pathways and phenotypes. Further association analysis of these molecules with other diseases should be carried out. If there’s a great overlap within other disorders, a panel of potential biomarkers can be developed.

By following a logic criterion, under statistical evaluation and through a completely unbiased approach, it was shown its assertiveness. It was possible to identify some consistent molecules among the studies that were previously associated with MS aetiology and new ones, as well as the identification of potential pathways associated with the disease, however it is evident that other important molecules and elements exist in the MS aetiology that could not be uncovered by the approach used here due to heterogeneity and other influencing factors. Through this analysis presented here, the results obtained in this study might give a starting point to establish traditional approaches by knowing in which processes to focus, opening a huge amount of possibilities to explore.

## Methods

### Dataset Identification

Human transcription profiles were identified and retrieved from the Gene Expression Omnibus (GEO) database^44^. ‘Multiple sclerosis’ and ‘Peripheral blood’ were used as key words for the first search and “miRNA” was used additionally for queries of miRNAs studies. A total of 18 datasets between gene transcription profiles (11) (GEO expression datasets GSE53716, GSE26484, GSE16461, GSE26104, GSE24427, GSE23205, GSE21942, GSE23832, GSE5574, GSE27688 and GSE43591) and miRNA transcription profiles (7) (GSE21079, GSE74579, GSE43590, GSE46282, GSE46283, GSE27690 and GSE17846) were obtained and further analysed through GEO2R. GEOR2 is a statistical package embedded in GEO that was used to compare groups of GEO datasets (e.g. healthy vs. disease). Two main groups across all datasets were analysed, namely healthy against disease and disease against disease treated. Comparative datasets for EBV infection (2 datasets) (GEO expression datasets GSE45918 (8 case and 8 control samples) and GSE45919 (15 case and 9 control samples)), inflammation data (3 datasets) derived from allergic reaction (GSE1964 (46 case and 37 control samples)), sacroidosis (GSE19314 (38 case and 20 control samples)) and TB infection (GSE28623 (46 case and 37 control samples), omitting non-symptomatic but TB positive patients) and autoimmune diseases (10 datasets) including SLE (GEO expression datasets GSE10325, GSE45923, GSE55447, GSE30153, GDS4185, GSE50395, GSE51997, GSE20864, GSE17755, GSE1964 (352 cases and 230 controls in total)), RA (GSE17755 and GSE1964 (120 cases and 61 controls)), JIA (GSE17755 (57 cases and 53 controls)) and ERA (GSE1964 (15 case and 8 control samples)) were identified and processed in a similar fashion.

GEOR2 performs a Student t-test statistical analysis (p-value calculation, as well as other output parameters such as fold-changes), generates tables of gene expression comparison between the chosen groups and sorts the molecules by significance. P-values were adjusted using Benjamini-Hochberg (BH) correction^45^. In case of a statistically poor dataset, where adjusted p-values were greater than 0.05, the unadjusted values were used.

### Data processing

All datasets were processed simultaneously. The molecules from the dataset comparisons were cross-mapped to the Pan-omics Analysis DataBase (PADB)^46^ in order to harmonise all data in a non-redundant fashion and for the purpose of cross-mapping to other databases (i.e. EnsEMBL, Uniprot, Gene names) using an in-house software (AWASH). Non-matched molecular identifiers were additionally covered using BioMart^47^.

Only molecules with a significant p-value of less than 0.05 for each comparison were kept. These molecules were subsequently used via further manual curation to develop a database named MuScle. The generated database is publicly available at www.PADB.org/muscle/ (See Supplementary Figure S1), it links to several external molecular identifiers (i.e. EnsEMBL, Uniprot/SwissProt, Gene ID, UniGene, OMIM) and to biomedical literature publications by PubMed identifiers. The database storages and integrates information regarding the experimental setup of the studies (sample characteristics, processing and detection), molecular information (such as external database identifiers, names, regulation in the disease and statistical scores) and links to the clinical information of the studies (cohort size, number of cases and controls, type of treatment administered and other clinical parameters).

All datasets were merged using the in-house AWASH software. Within each dataset, the repeated molecules were either averaged or removed when fold-change values were observe to be oppositely regulated. Further thresholding involved a cut-off value of a log 2 fold change (log2FC) with a log2FC >1 and <−1.

We carried out dimensionality reduction through Principal Component Analysis (PCA) using Multibase (Numerical Dynamics, Japan, 2015) to enable filtering, such as outlier removal, and rearrange or remove samples based on patterns of gene expression across all the datasets comparisons. After plotting the variables (loadings), inconsistent datasets were removed and the remaining datasets were split into the distinct groups based on the PCA. The first includes all the dataset comparisons (“General” group), the second includes the dataset comparisons that fitted the healthy (control) versus disease group (“Non-treated” group) and the third handles dataset comparisons that fitted the disease treated (control) versus disease group (“Treated” group).

### Functional analyses

Functional analyses of the three groups were carried out. Gene ontology (GO) and pathway term clustering using ClueGO v2.4 and CluePedia version 1.5, and the Cytoscape network analysis framework were performed. The default parameters were used with an overall statistical significance value set to p-value < 0.05.

From the total molecules a frequency threshold was applied, keeping as the most significant the ones above 20% of frequency among the datasets (Table 3). For each of the groups created (general, treated, non-treated) several ClueGO analyses were performed, each of it with wider thresholds. GO analysis for biological processes was performed and pathways were determined by mapping the molecules tagged into WikiPathways.

**Table 3:** Number of molecules per threshold applied for each group.

Threshold	Group G	Group HD	Group DD
1ST	37 (>20%)	19 (>23%)	72 (>23%)
2ND	52 (>17%)	66 (>15%)	93 (>18%)
3RD	72 (>14%)		134 (>14%)
4TH	97 (>11%)		

Group G consists of all molecules across all samples, Group HD is based on the comparison between healthy and MS patients (“untreated”) and Group DD consists of MS samples and samples from treated patients (“treated”). The threshold value corresponds to frequency % of molecules observed in specific groups and is shown in brackets after the number of molecules in each group.

### Pathway analyses

Visualisation and pathway statistical inference was carried out in PathVisio 3 and the human collection of pathways from WikiPathways. The most significant molecules were imported into PathVisio and pathway statistical analysis was performed. First, the most significant molecules were analysed by making a table where the EnsEMBL ID, frequency (absolute values) and logFC were included. Once imported into Pathvisio, statistical analyses were done and the molecules were mapped; a specific threshold value for frequency and logFC needed to be entered for the analyses to begin. For visualization, the options were changed into a colour gradient from red to green, from 2 to −2 as for logFC values.

This primary analysis in PathVisio was done in order to identify and compare the most significant pathways with the analysis results of the pathway term clustering from ClueGO. Once the pathways were identified and converging on both analysis tools, all the significant molecules were added into the PathVisio software in order to fill the gaps of these pathways.

**Figure 9:**
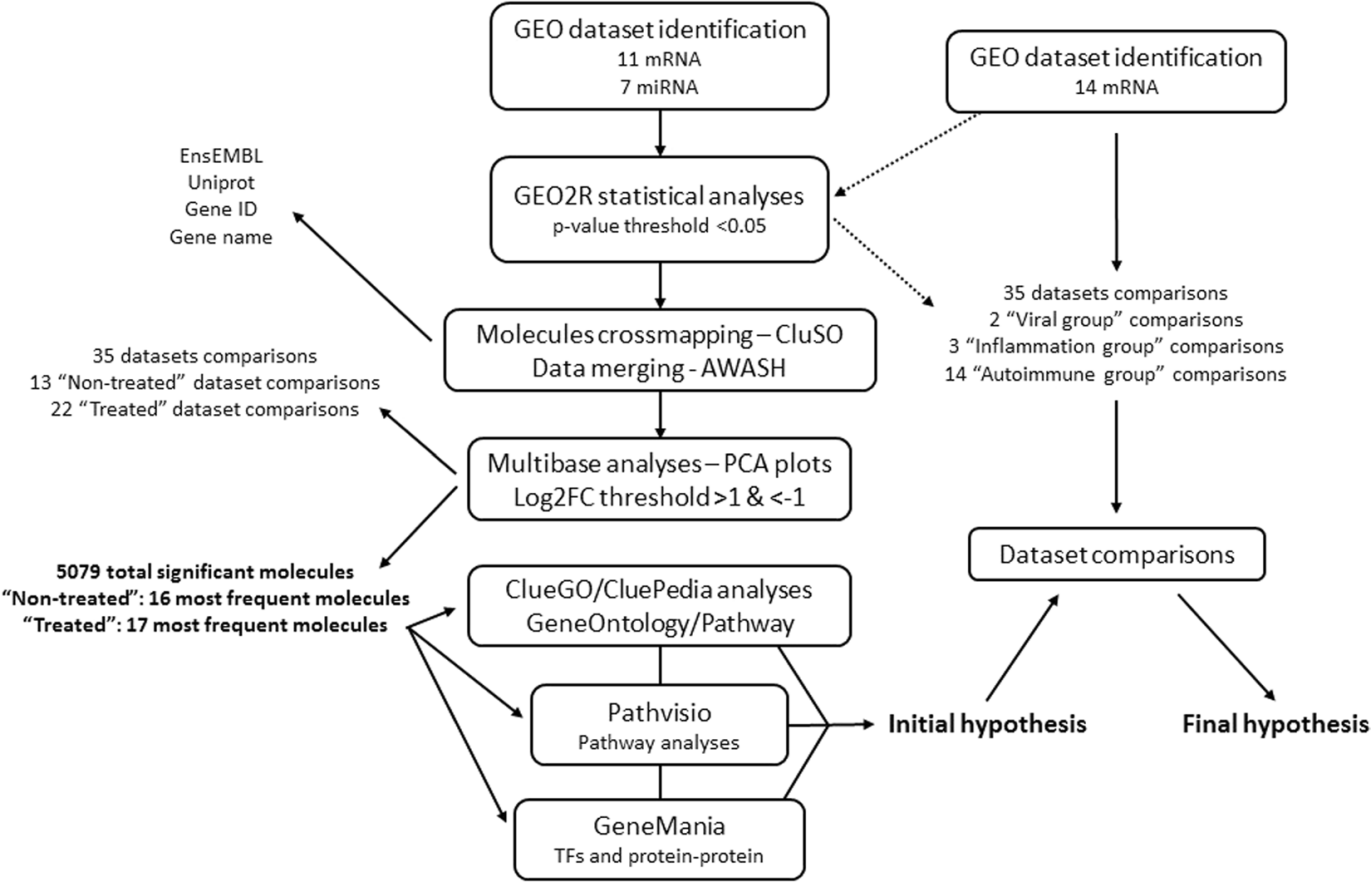
Summary of the methodology followed in this study. The arrows depict the flow of the various steps. ClueGO/CluePedia, PathVisio and GeneMania analysis results were merged in order to generate the initial and final hypothesis.

To enrich and generate the final original MS pathways, GeneMania was used to display protein–protein interactions (PPI’s) and to identify regulatory elements such as transcription factors (TF’s) across the most significant molecules. The results of the several bioinformatics iterations used in the study workflow (Fig. 9) were combined and a final hypothesis was generated using validation by deep-mining of the literature. The description and citation of the previous tools and software solutions used in this integrative systems biology pipeline can be found elsewhere^48^, otherwise they are cited within text.

## Electronic supplementary material

Supplementary information
