# Supplementary material for: Integrative analysis of Multiple Sclerosis using a systems biology approach
Source: Sci Rep. 2018 Apr 4;8:5633. doi: 10.1038/s41598-018-24032-8 (PMC5884799; doi:10.1038/s41598-018-24032-8)
Supplement: Supplementary file 1 — Supplementary information [file 41598_2018_24032_MOESM1_ESM.pdf]

Integrative analysis of Multiple Sclerosis using a systems biology approach

Supplementary data

Karla Cervantes-Gracia[1] and Holger Husi[2,3*]

[1]University of Monterrey, Health Sciences Division, Monterrey, 66238, Mexico.

[2]Institute of Cardiovascular and Medical Sciences, BHF Glasgow Cardiovascular Research Centre, University of Glasgow, Glasgow, G12 8TA, UK

[3]Department of Diabetes and Cardiovascular Science, University of the Highlands and Islands, Centre for Health Science, Inverness IV2 3JH, UK

[*]Holger.Husi@glasgow.ac.uk

Table S1 Description of gene expression profile studies included in this work.

GEO-ID	EXPREF	PMID	Profile	Groups	Case	Control	Disease case	Disease control	Treatment	Tissue	Subcell	Platform
GSE53716	Exp24850724	24850724	mRNA	12	6	6	CIS	Baseline	IFN-β-1a	Blood		Affymetrix GPL570
GSE26484	Exp22491253	22491253	mRNA	10	6	4	RRMS	Healthy		Blood		Affymetrix GPL570
GSE16461	Exp21216829	21216829	mRNA	8	4	4	RRMS/SPMS	Healthy		Blood	CD4+/CD8+	Affymetrix GPL570
GSE26104	Exp21886806	21886806	mRNA	32	24	8	RRMS	Baseline	IFN-β-1b/1a	Blood		Affymetrix GPL570
GSE24427	Exp20136355	20136355	mRNA	125	100	25	RRMS	RRMS	rIFN-β-1b	Blood		Affymetrix GPL96 & GPL97
GSE23205	Exp20921521	20921521	mRNA	10	6	4	PPMS/Risk haplotype	PPMS/Protective haplotype		Blood		Affymetrix GPL570
GSE21942	Exp22021740	22021740	mRNA	27	12	15	MS	Healthy		Blood		Affymetrix GPL570
GSE23832	Exp21346816	21346816	mRNA	12	8	4	MS	Healthy		Blood		Affymetrix GPL6244
GSE5574	Exp17467740	17467740	mRNA	15	10	5	RRMS	RRMS	IFN-β-1a	Blood		GE Healthcare GPL4191
GSE27688	Exp22252466A	22252466	mRNA	26	16	10	RRMS/SPMS	Healthy	aHSCT	Blood	CD34+/CD34-	Agilent GPL6480
GSE43591	Exp23895517A	23895517	mRNA	19	10	9	RRMS	Healthy		Blood	T-cells	Affymetrix GPL570
GSE21079	Exp20711463	20711463	miRNA	96	59	37	RRMS/SPMS/PPMS	Healthy		Blood		Illumina GPL8178
GSE74579	Exp26831009	26831009	miRNA	72	48	24	RRMS	Healthy		Blood		Affymetrix GPL8786
GSE43590	Exp23895517B	23895517	miRNA	20	11	9	RRMS	Healthy	IFN-β	Blood		Affymetrix GPL14613
GSE46282	Exp23921681B	23921681	miRNA	24	18	6	RRMS/CIS	Baseline	IFN-β-1b	Blood		TaqMan GPL13328
GSE46283	Exp23921681C	23921681	miRNA	6	3	3	RRMS/CIS	Baseline	IFN-β-1b	Blood		Affymetrix GPL14613
GSE27690	Exp22252466B	22252466	miRNA	9	4	5	SPMS	Healthy	aHSCT	Blood	CD34+	Agilent GPL10850
GSE17846	Exp19823682	19823682	miRNA	41	20	21	RRMS	Healthy		Blood		Febit GPL9040

Table S2 Gene expression profile studies - Dataset groups characteristics.

DEMOGRAPHREF	EXPREF	Group Size	Treatment	Group classifier	Label	Gender
Dem24850724a	Exp24850724	6	IFN-β-1a	CIS	Before treatment (baseline)	
Dem24850724b	Exp24850724	6	IFN-β-1a	CIS	365 days follow-up	
Dem22491253a	Exp22491253	4		Healthy	Control	
Dem22491253b	Exp22491253	3		RRMS	High Sema4A	
Dem22491253c	Exp22491253	3		RRMS	Low Sema4A	
Dem21216829a	Exp21216829	4		Healthy	CD4+ (Control monozigotic twin)	2/6 (M/F)
Dem21216829b	Exp21216829	4		Healthy	CD8+ (Control monozigotic twin)	2/6 (M/F)
Dem21216829c	Exp21216829	4		RRMS/SPMS	CD4+ (3 RRMS/1 SPMS monozigotic twin)	2/6 (M/F)
Dem21216829d	Exp21216829	4		RRMS/SPMS	CD8+ (3 RRMS/1 SPMS monozigotic twin)	2/6 (M/F)
Dem21886806a	Exp21886806	8	IFN-β-1b/1a	RRMS	Before treatment (baseline)	0/8 (M/F)
Dem21886806b	Exp21886806	4	IFN-β-1b	RRMS	91 days follow-up	0/4 (M/F)
Dem21886806c	Exp21886806	4	IFN-β-1b	RRMS	365 days follow-up	0/4 (M/F)
Dem21886806d	Exp21886806	4	IFN-β-1b	RRMS	730 days follow-up	0/4 (M/F)
Dem21886806e	Exp21886806	4	IFN-β-1a	RRMS	91 days follow-up	0/4 (M/F)
Dem21886806f	Exp21886806	4	IFN-β-1a	RRMS	365 days follow-up	0/4 (M/F)
Dem21886806g	Exp21886806	4	IFN-β-1a	RRMS	730 days follow-up	0/4 (M/F)
Dem20136355a	Exp20136355	25	rIFN-β-1b	RRMS	Before treatment (baseline)	9/16 (M/F)
Dem20136355b	Exp20136355	25	rIFN-β-1b	RRMS	2 days follow-up	9/16 (M/F)
Dem20136355c	Exp20136355	25	rIFN-β-1b	RRMS	30 days follow-up	9/16 (M/F)
Dem20136355d	Exp20136355	25	rIFN-β-1b	RRMS	365 days follow-up	9/16 (M/F)
Dem20136355e	Exp20136355	25	rIFN-β-1b	RRMS	730 days follow-up	9/16 (M/F)
Dem20136355aa	Exp20136355	25	rIFN-β-1b	RRMS	Before treatment (baseline)	9/16 (M/F)
Dem20136355bb	Exp20136355	25	rIFN-β-1b	RRMS	2 days follow-up	9/16 (M/F)
Dem20136355cc	Exp20136355	25	rIFN-β-1b	RRMS	30 days follow-up	9/16 (M/F)
Dem20136355dd	Exp20136355	25	rIFN-β-1b	RRMS	365 days follow-up	9/16 (M/F)
Dem20136355ee	Exp20136355	25	rIFN-β-1b	RRMS	730 days follow-up	9/16 (M/F)
Dem20921521a	Exp20921521	4		PPMS	PRF1 gene/Protective haplotype	4/0 (M/F)
Dem20921521b	Exp20921521	6		PPMS	PRF1 gene/Risk haplotype	6/0 (M/F)
Dem22021740a	Exp22021740	15		Healthy	Control	0/15 (M/F)

Dem22021740b	Exp22021740	12		MS	Case	0/12 (M/F)
Dem21346816a	Exp21346816	4		Healthy	Control	
Dem21346816b	Exp21346816	8		MS	Case	
Dem17467740a	Exp17467740	5	IFN-β-1a	RRMS	Before treatment (baseline)	
Dem17467740b	Exp17467740	5	IFN-β-1a	RRMS	1 days follow-up	
Dem17467740c	Exp17467740	5	IFN-β-1a	RRMS	182 days follow-up	
Dem23921681Ba	Exp23921681B	6	IFN-β-1b	RRMS/CIS	Before treatment (baseline)	0/6 (M/F)
Dem23921681Bb	Exp23921681B	6	IFN-β-1b	RRMS/CIS	First inyection follow-up	0/6 (M/F)
Dem23921681Bc	Exp23921681B	6	IFN-β-1b	RRMS/CIS	Second inyection follow-up	0/6 (M/F)
Dem23921681Bd	Exp23921681B	6	IFN-β-1b	RRMS/CIS	30 days follow-up	0/6 (M/F)
Dem23921681Ca	Exp23921681C	3	IFN-β-1b	RRMS/CIS	Before treatment (baseline)	0/3 (M/F)
Dem23921681Cb	Exp23921681C	3	IFN-β-1b	RRMS/CIS	30 days follow-up	0/3 (M/F)
Dem22252466Aa	Exp22252466A	5		Healthy	CD34+ HPC Control	2/3 (M/F)
Dem22252466Ab	Exp22252466A	8		RRMS/SPMS	CD34+ HPC	2/6 (M/F)
Dem22252466Ac	Exp22252466A	5		Healthy	CD34- HPC Control	2/3 (M/F)
Dem22252466Ad	Exp22252466A	8		RRMS/SPMS	CD34- HPC	2/6 (M/F)
Dem22252466Ba	Exp22252466B	5		Healthy	CD34+ HPC Control	2/3 (M/F)
Dem22252466Bb	Exp22252466B	4		MS	CD34+ HPC	0/4 (M/F)
Dem23895517Aa	Exp23895517A	9		Healthy	T-cells Control	4/5 (M/F)
Dem23895517Ab	Exp23895517A	10		RRMS	T-cells	6/4 (M/F)
Dem23895517Ba	Exp23895517B	9		Healthy	Control	4/5 (M/F)
Dem23895517Bb	Exp23895517B	11	IFN-β	RRMS	Case	1/10 (M/F)
Dem20711463a	Exp20711463	37		Healthy	Control	
Dem20711463b	Exp20711463	24		RRMS	Case	
Dem20711463c	Exp20711463	17		SPMS	Case	
Dem20711463d	Exp20711463	18		PPMS	Case	
Dem26831009a	Exp26831009	24		Healthy	Control	10/14 (M/F)
Dem26831009b	Exp26831009	24		RRMS	Relapse stage	10/14 (M/F)
Dem26831009c	Exp26831009	24		RRMS	Remission stage	10/14 (M/F)
Dem19823682a	Exp19823682	21		Healthy	Control	
Dem19823682b	Exp19823682	20	IFN-β or Glatiramer acetate	RRMS	Case	

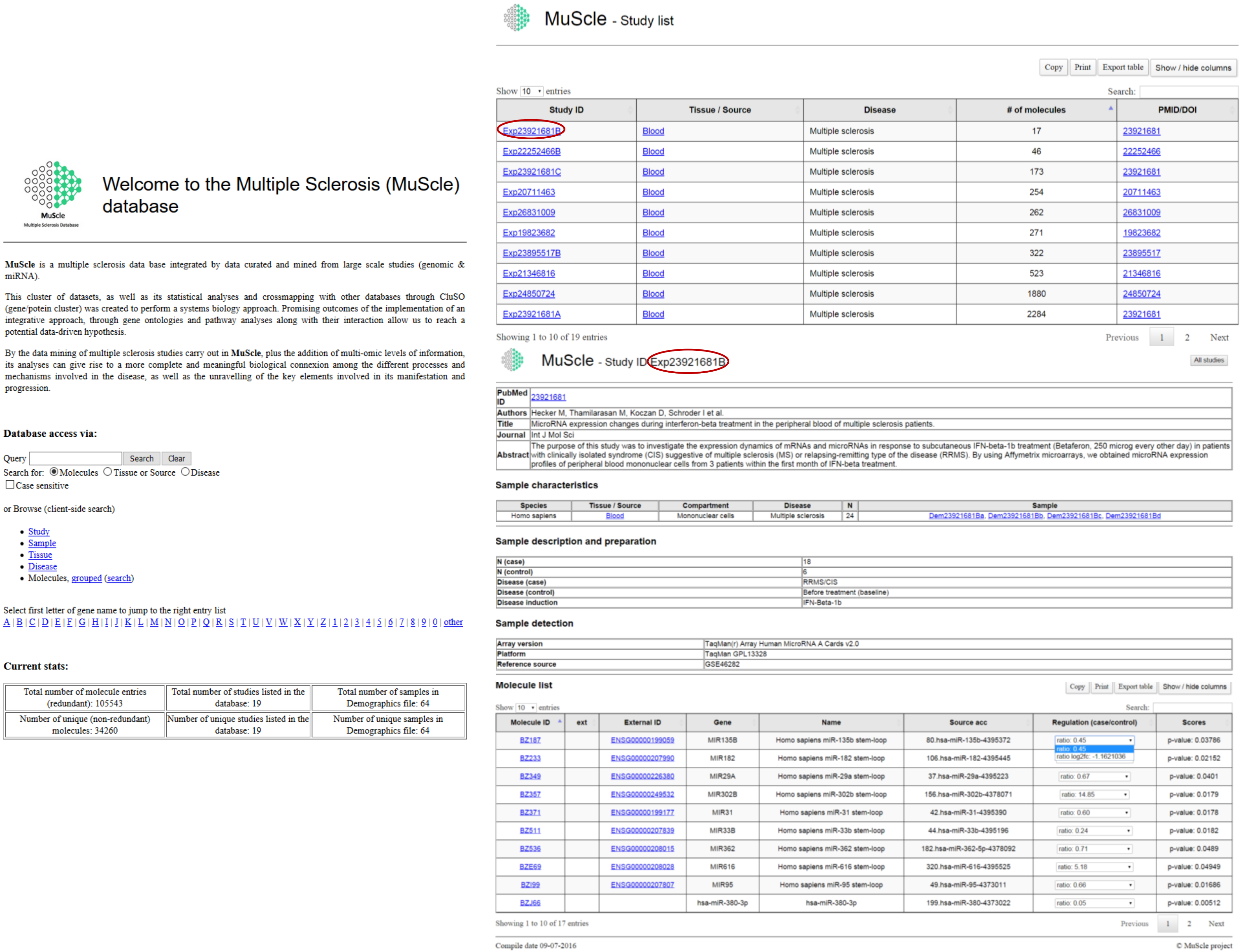

Figure S1 Page-views of the multiple sclerosis database (MuScle). The database is organised and can be accessed by type of study (the study “Exp23921681B” is highlighted in red as an example how the information of the study is stored and displayed in the MuScle database), sample (handles clinical information), tissue/fluid source and by specific queries of molecules. Available at http://www.padb.org/muscle/.
